# LncRNA TTN-AS1 acts as a tumor promoter in gallbladder carcinoma by regulating miR-107/HMGA1 axis

**DOI:** 10.1186/s12957-021-02279-2

**Published:** 2021-06-05

**Authors:** Zhaoxia Lin, Yaosheng Li, Rongfeng Shao, Yuqing Hu, Han Gao

**Affiliations:** 1 Department of Clinical Laboratory, Jinan Central Hospital, Cheeloo College of Medicine, Shandong University, Shandong Province, Jinan, 250013 China; 2 Imaging Department, The People’s Hospital of Zhangqiu Area, Shandong Province, Jinan, 250200 China; 3 Department of Hepatobiliary Vascular Surgery, Qingdao Central Hospital, Qingdao University, Shandong Province, Qingdao, 266000 China; 4 Department of Endocrinology, The People’s Hospital of Zhangqiu Area, Shandong Province, Jinan, 250200 China; 5 Department of Pathology, Qingdao Municipal Hospital, No. 5 Donghai Middle Road, Shandong Province, Qingdao, 266071 China

**Keywords:** TTN-AS1, Gallbladder carcinoma, miR-107, HMGA1

## Abstract

**Background:**

The incidence of gallbladder carcinoma (GBM) in China has increased in recent years. Here, the functional mechanism of lncRNA TTN-AS1 in GBC was preliminary elucidated.

**Methods:**

The expression levels of lncRNA TTN-AS1, miR-107, and HMGA1 in tissues and cell lines were assessed by RT-qPCR. Cell proliferation was measured by MTT assays. Cell invasion and migration abilities were evaluated by Transwell assays. The relationship between miR-107 and lncRNA TTN-AS1 or HMGA1 was confirmed by luciferase reporter assay.

**Results:**

Upregulation of lncRNA TTN-AS1 and downregulation of miR-107 were detected in GBC. Furthermore, the expressions between TTN-AS1 and miR-107 were mutually inhibited in GBC. Functionally, lncRNA TTN-AS1 promoted cell viability and motility in GBC by sponging miR-107. In addition, miR-107 directly targets HMGA1. And HMGA1 can be positively regulated by lncRNA TTN-AS1 in GBC. Furthermore, HMGA1 promoted GBC progression by interacting with lncRNA TTN-AS1/miR-107 axis.

**Conclusion:**

LncRNA TTN-AS1 acted as a tumor promoter in GBC by sponging miR-107 and upregulating HMGA1.

## Background

Gallbladder carcinoma (GBC) refers to a malignant tumor that originates in the epithelial tissue of the gallbladder, accounting for 5% of the entire digestive tract tumor and 1% of the systemic malignant tumor [1, 2]. The etiology of GBC is obscure and may be related to gallstones [3]. GBC usually occurs more frequently in women than in men and is more common in people around the age of 50–70 [4]. It is a disease with a high degree of malignancy and a poor treatment effect [5, 6]. Most of GBC patients seen clinically are advanced, and the rate of radical resection is low [7]. The 1-year survival rate in GBC patients is less than 80% and the 5-year survival rate is less than 5% [8]. Moreover, because early symptoms of GBC are not obvious, the early diagnosis rate is low [9]. Therefore, it is important to explore new molecular markers for early diagnosis of GBC.

Previous studies have demonstrated that long noncoding RNAs (lncRNAs) exert important role in the onset of cancer, including GBC. For instance, lncRNA DILC was upregulated in GBC, and promoted GBC progression [10]. And upregulation of H19 indicated a poor prognosis in GBC and promoted epithelial-mesenchymal transition (EMT) [11]. The dysregulation of lncRNA TTN antisense RNA 1 (TTN-AS1) has been found in malignancies. High expression of lncRNA TTN-AS1 has been detected in lung adenocarcinoma and osteosarcoma [12, 13]. Functionally, lncRNA TTN-AS1 was found to promote cell proliferation and inhibit apoptosis in prostatic cancer by sponging miR-193a-5p [14]. In addition, lncRNA TTN-AS1 was proposed to promote migration, invasion, and EMT of lung adenocarcinoma via sponging miR-142-5p [15]. Yet the possible mechanism of TTNAS1 has not been explored in GBC. Those researches reveal that lncRNA TTN-AS1 regulates tumorigenesis by binding to some microRNAs (miRNAs).

Here, miR-107 was found to have a binding site with lncRNA TTN-AS1. Moreover, miR-107 has been demonstrated to be involved in human cancers. Upregulation of miR-107 has been identified in colon cancer and hepatocellular carcinoma [16, 17]. Deregulated expression of miR-107 was found to inhibit metastasis of pancreatic ductal adenocarcinoma [18]. However, miR-107 expression was decreased in prostate cancer and ovarian cancer [19, 20]. MiR-107 was also demonstrated to inhibit gastric cancer cell proliferation and metastasis [21]. These results suggest that miR-107 expression and function are tissue specific. Additionally, lncRNA FOXC2-AC1 was proposed to promote lung cancer metastasis by regulating miR-107 [22]. But the interaction between lncRNA TTN-AS1 and miR-107 in GBC remains unclear.

In addition, high-mobility group AT-hook1 (HMGA1) was predicted to be a possible target of miR-107. The specific role of HMGA1 was also reported in previous studies. HMGA1 was upregulated in osteosarcoma, cervical, and colorectal cancer [23, 24]. And HMGA1 exacerbated tumor growth and accelerated migration/invasion in cervical cancer [25]. Moreover, miR-625 has been revealed to suppress cell proliferation and migration by targeting HMGA1 in breast cancer [26]. However, the regulatory mechanism of HMGA1/miR-107/lncRNA TTN-AS1 is poorly illuminated in GBC. In our research, we aimed to preliminarily elucidate the function as well as the functional mechanism of lncRNA TTN-AS1 in GBC.

## Methods

### Clinical tissues

Thirty-eight GBC tissues and paired normal tissues were obtained from Qingdao Municipal Hospital. These GBC patients signed the informed consents. The procedure of this study was approved by the Institutional Ethics Committee of Qingdao Municipal Hospital.

### Cell lines and culture

GBC-SD cells were obtained from BeNa Culture Collection (BNCC, Beijing, China). The culture condition of GBC-SD cells include Dulbecco’s modified Eagle’s medium (DMEM), 10% FBS, 5% CO_2_, and 37 °C.

### Cell transfection

TTN-AS1 plasmid or siRNA, miR-107 mimics or inhibitor, and HMGA1 siRNA were obtained from RiBoBio (Guangzhou, China). Next, Lipofectamine 2000 (Invitrogen/Thermo Fisher Scientific) was applied to transfect them into GBC-SD cells.

### RNA isolation, reverse transcription, and RT-qPCR

TRIzol reagent (TaKaRa, Dalian, China) was used to extract total RNA. The complementary DNA (cDNA) was synthesized by Reverse Transcription Kit (TaKaRa). Real-time PCR Mixture assays (TaKaRa) and primers were used to perform RT-qPCR assay. The internal controls were *18S* rRNA and U6. The relative expression of mRNAs was quantified with the 2^−△△ct^ method. The primers used were the following: TTN-AS1 forward 5′-CGG GAA CAA GCC CTG TG-3′; TTN-AS1 reverse 5′-CCG GCC CAA AGA TGA TG-3′; miR-107 forward: 5′-AGC AGC AUU GUA CAG GGC UAU CA-3′ and reverse, 5′-CGC AAG GAT GAC ACC AAA TTC-3′; U6-forward: 5′-GCT TCG GCA GCA CAT ATA CTA AAA T-3′ and reverse, 5′-CGC TTC ACG AAT TTG CGT GTC AT-3′; HMGA1 forward: 5′-GCT GGT AGG GAG TCA GAA GGA-3′ and reverse, 5′-TGG TGG TTT TCC GGG TCT TG-3′; Human *18S* rRNA, forward: 5′-AGA AAC GGC TAC CAC ATC CA-3′, and reverse: 5′- CAC CAG ACT TGC CCT CCA-3′.

### MTT assay

MTT solution was used to incubate transfected GBC-SD cells to detect cell viability. The absorbance at 490 nm detected by a microscope (Olympus Corp, Tokyo, Japan) assessed cell proliferation.

### Transwell assay

Cell invasion and migration were examined in the upper chamber with or without Matrigel. The experimental procedure was performed according to previous study [6]. Observation and photographing were performed by a light microscope.

### Dual-luciferase reporter assay

The pmiR-GLO vectors (Promega, Beijing, China) containing 3′-UTR of wild-type and mutant TTN-AS1 or HMGA1 and miR-107 mimics were transfected into GBC-SD cells. After 48 h, luciferase activities were determined by dual-luciferase reporter assay system (Promega).

### Statistical analysis

Data are shown as mean ± SD. Differences were analyzed using Student’s t-test or one-way ANOVA in SPSS 19.0 or Graphpad Prism 6 software. P < 0.05 indicates statistical significance.

## Results

### LncRNA TTN-AS1 and miR-107 express abnormally in GBC

**Fig. 1:**
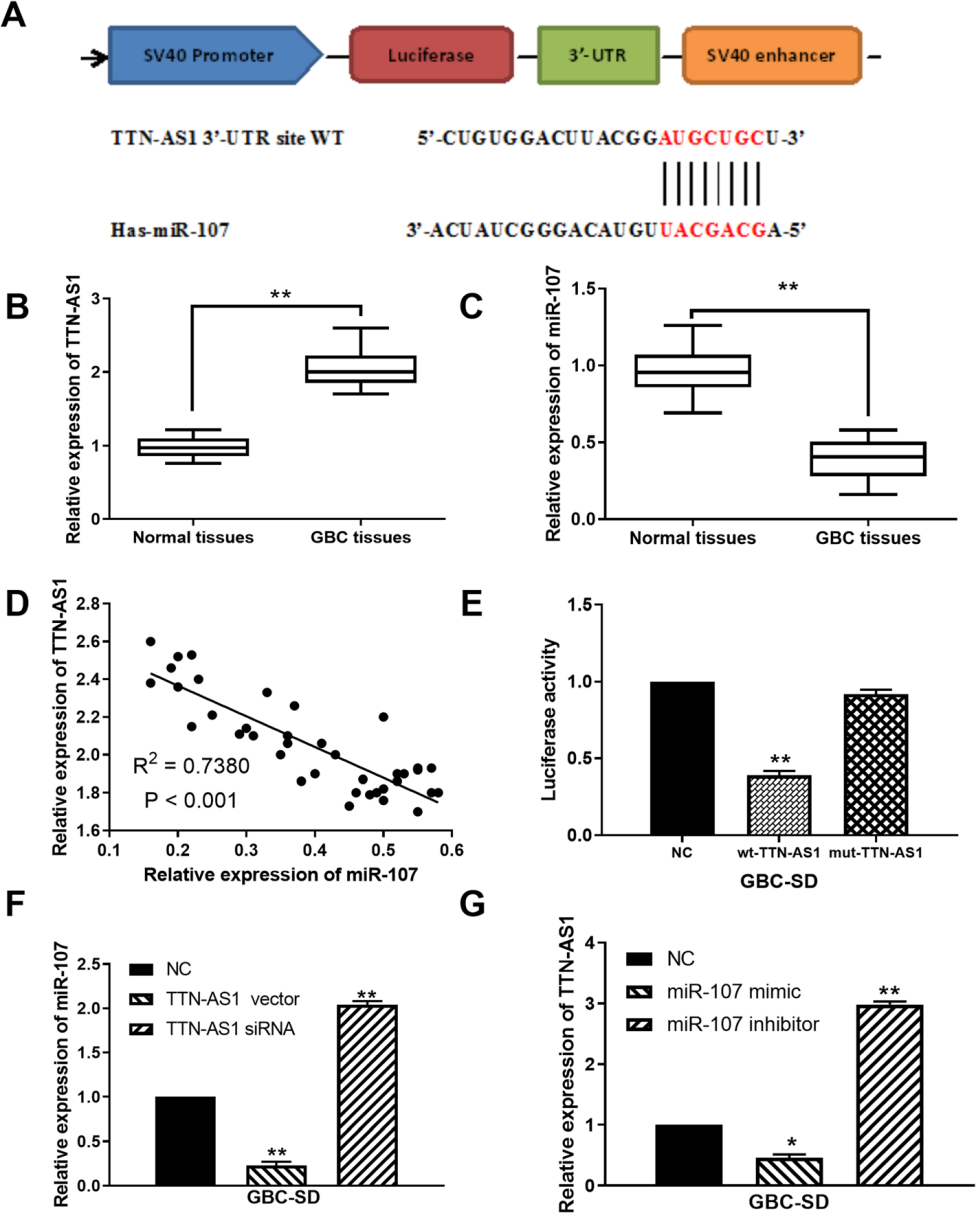
LncRNA TTN-AS1 and miR-107 express abnormally in GBC. **A** The binding sites between TTN-AS1 with miR-107. **B** TTN-AS1 expression in GBC tissues and normal tissues. **C** MiR-107 expression in GBC tissues and normal tissues. **D** MiR-107 was negatively correlated with TTN-AS1 expression in GBC tissues. **E** Luciferase reporter assay. **F** MiR-107 expression regulated by TTN-AS1 siRNA or vector in GBC-SD cells. **G** TTN-AS1 expression in GBC-SD cells containing miR-107 mimics or inhibitor.*P < 0.05, **P < 0.01

Based on the prediction of starBase database (http://starbase.sysu.edu.cn/), lncRNA TTN-AS1 was found to have a binding site with miR-107 (Fig. 1A). Then, lncRNA TTN-AS1 was found to be upregulated in GBC tissues compared to normal tissues (Fig. 1B). On the contrary, miR-107 expression was decreased in GBC tissues in contrast to normal tissues (Fig. 1C). Meanwhile, we found that lncRNA TTN-AS1 was negatively correlated with miR-107 expression in GBC tissues (Fig. 1D). Next, dual luciferase reporter was used to further verify their relationship. The result indicated that miR-107 mimics reduced the luciferase activity of wt-TTN-AS1 but had little effect on mut-TTN-AS1 in GBC-SD cells (Fig. 1E). In addition, RT-qPCR suggested that TTN-AS1 upregulation decreased miR-107 expression, whereas TTN-AS1 downregulation promoted miR-107 expression in GBC-SD cells (Fig. 1F). However, TTN-AS1 expression was also reduced by miR-107 mimics and enhanced by miR-107 inhibitor in GBC-SD cells (Fig. 1G). These results imply that lncRNA TTN-AS1 and miR-107 express abnormally in GBC. Furthermore, the expressions between TTN-AS1 and miR-107 are mutually inhibited in GBC.

### LncRNA TTN-AS1/miR-107 axis is involved in GBC progression

**Fig. 2:**
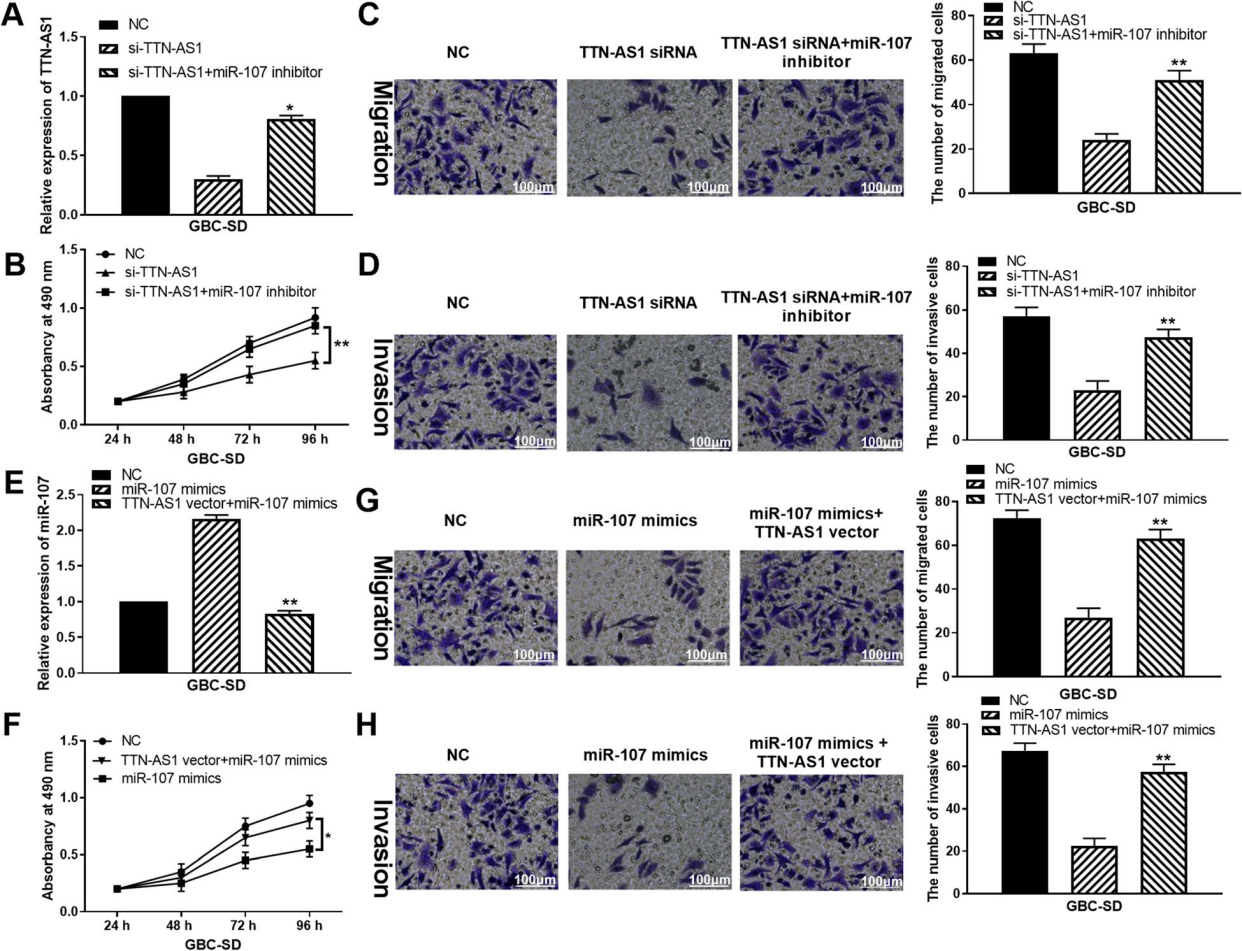
LncRNA TTN-AS1/miR-107 axis is involved in GBC progression. **A** TTN-AS1 expression in GBC-SD cells with TTN-AS1 siRNA or TTN-AS1+miR-107 inhibitor. **B**–**D** Cell proliferation, migration, and invasion in GBC-SD cells with TTN-AS1 siRNA or TTN-AS1+miR-107 inhibitor. **E** MiR-107 expression in GBC-SD cells with miR-107 mimics or miR-107 mimics+TTN-AS1 vector. **F**–**H** Cell proliferation, migration, and invasion in GBC-SD cells with miR-107 mimics or miR-107 mimics+TTN-AS1 vector. *P < 0.05, **P < 0.01

To explore the regulatory mechanism of lncRNA TTN-AS1/miR-107, TTN-AS1 siRNA or TTN-AS1 siRNA+miR-107 inhibitor was transfected into GBC-SD cells. TTN-AS1 expression was declined by its siRNA, but miR-107 inhibitor recovered this reduction of TTN-AS1 expression (Fig. 2A). MTT assay showed that TTN-AS1 downregulation restrained cell proliferation in GBC-SD cells, while miR-107 inhibitor reversed its inhibitory effect on cell proliferation (Fig. 2B). Additionally, Transwell assay displayed that GBC-SD cell migration and invasion were inhibited by TTN-AS1 downregulation. MiR-107 downregulation restored this inhibition of cell migration and invasion (Fig. 2C, D). Then, GBC-SD cells with miR-107 mimics or miR-107 mimics+TTN-AS1 vector were used to explore the function of miR-107 in GBC. MiR-107 mimics were found to increase its expression, whereas this increased expression was recovered by TTN-AS1 upregulation (Fig. 2E). Additionally, miR-107 overexpression restrained cell proliferation in GBC-SD cells. TTN-AS1 upregulation also abolished the inhibitory effect of miR-107 on cell proliferation (Fig. 2F). Meanwhile, miR-107 overexpression played an inhibitory effect on GBC-SD cell migration and invasion. The reverse effect of TTN-AS1 vector on cell migration and invasion was also identified in GBC-SD cells (Fig. 2G, H). Collectively, lncRNA TTN-AS1 promoted cell viability and motility in GBC by sponging miR-107.

### HMGA1 is a direct target of miR-107

**Fig. 3:**
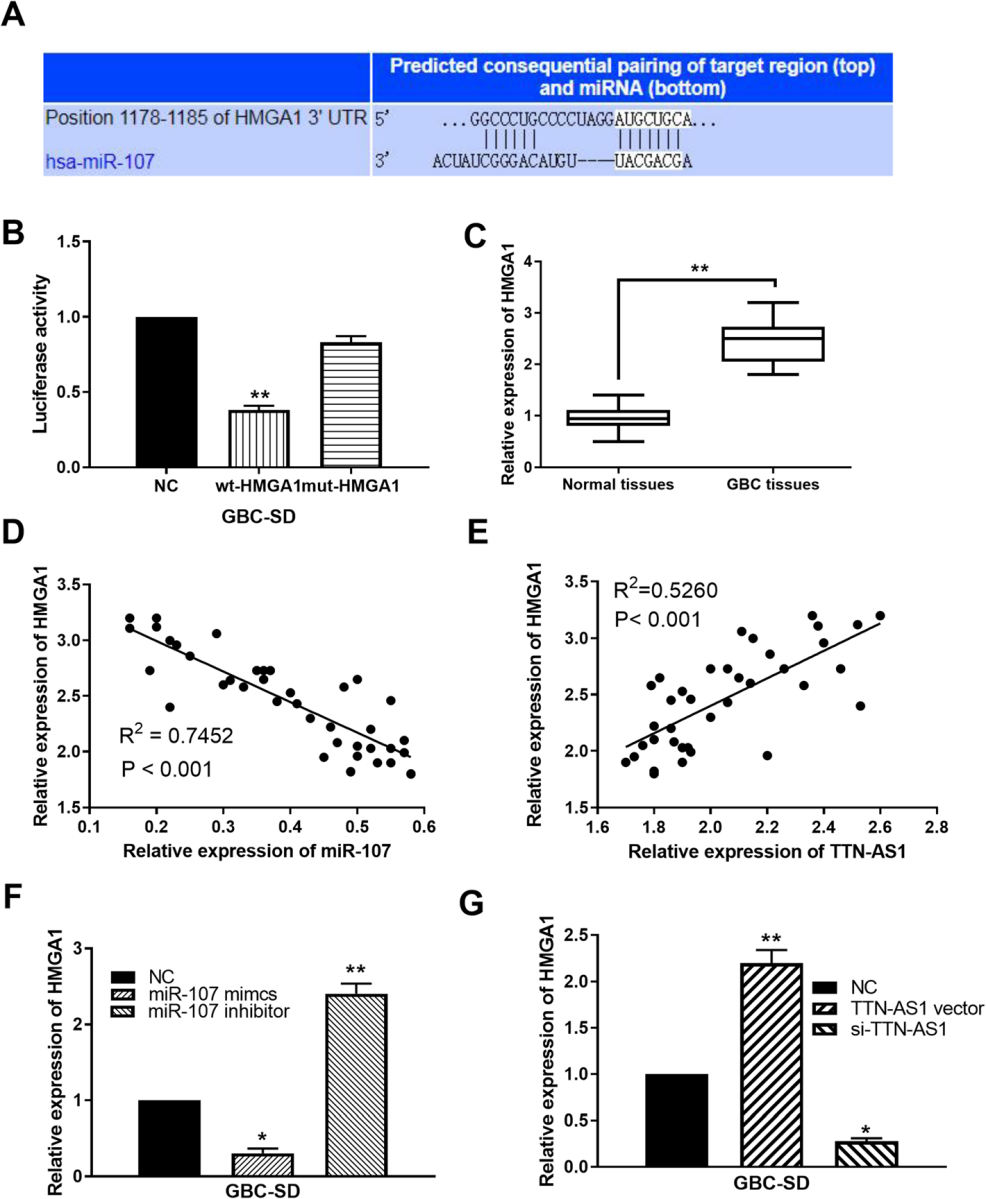
HMGA1 is a direct target of miR-107. **A** The binding sites between HMGA1 and miR-107. **B** Luciferase reporter assay. **C** HMGA1 expression in GBC tissues and normal tissues. **D** MiR-107 expression was negatively correlated with HMGA1 in GBC tissues. **E** TTN-AS1 was positively correlated with HMGA1 in GBC tissues. **F**, **G** HMGA1 expression regulated by miR-107 or TTN-AS1 in GBC-SD cells. *P < 0.05, **P < 0.01

To further elucidate the functional mechanism of miR-107, the downstream target of miR-107 was investigated. TargetScan database (http://www.targetscan.org) predicts that miR-107 can bind to 3′-UTR of HMGA1 (Fig. 3A). Luciferase reporter assay showed that miR-107 mimics declined the luciferase activity of wt-HMGA1 but had no effect on mut-HMGA1 (Fig. 3B). Additionally, HMGA1 was found to be upregulated in GBM tissues compared to normal tissues (Fig. 3C). At the same time, HMGA1 expression was inversely regulated by miR-107 expression in GBC tissues (Fig. 3D). On the contrary, we also found that HMGA1 expression was positively correlated with TTN-AS1 in GBC tissues (Fig. 3E). In GBC-SD cells, HMGA1 expression was reduced by miR-107 mimics and promoted by miR-107 inhibitor (Fig. 3F). Moreover, TTN-AS1 upregulation increased HMGA1 expression, while TTN-AS1 downregulation reduced HMGA1 expression in GBC-SD cells (Fig. 3G). These results reveal that miR-107 directly targets HMGA1. And HMGA1 can be positively regulated by lncRNA TTN-AS1 in GBC.

### HMGA1 participates in GBC development by interacting with lncRNA TTN-AS1/miR-107 axis

**Fig. 4:**
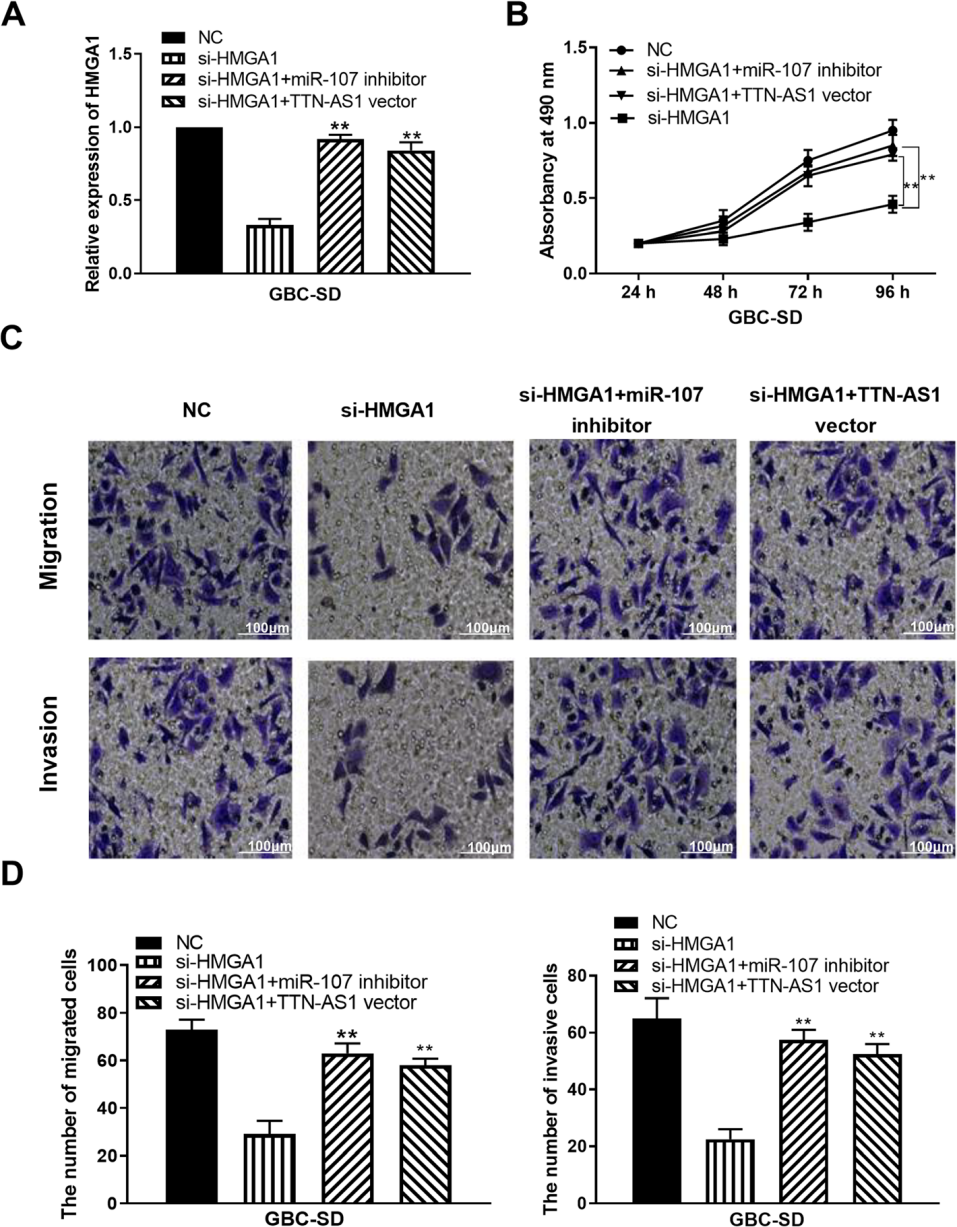
HMGA1 participates in GBC development by interacting with lncRNA TTN-AS1/miR-107 axis. **A** HMGA1 expression in GBC-SD cells with HMGA1 siRNA, HMGA1 siRNA+miR-107 inhibitor, or HMGA1 siRNA+TTN-AS1 vector. **B**–**D** Cell proliferation, migration, and invasion in GBC-SD cells with HMGA1 siRNA, HMGA1 siRNA+miR-107 inhibitor, or HMGA1 siRNA+TTN-AS1 vector. **P < 0.01

To elucidate the regulatory mechanism of lncRNA TTN-AS1/miR-107/HMGA1 axis, TTN-AS1 vector or miR-107 inhibitor was transfected into GBC-SD cells with HMGA1 siRNA. HMGA1 siRNA was found to downregulate its expression in GBC-SD cells. TTN-AS1 vector or miR-107 inhibitor restored the downregulation of HMGA1 (Fig. 4A). Functionally, TTN-AS1 upregulation or miR-107 downregulation abolished the inhibition of cell proliferation induced by HMGA1 siRNA (Fig. 4B). In addition, TTN-AS1 vector or miR-107 inhibitor abolished the inhibitory effect of HMGA1 siRNA on GBC-SD cell migration and invasion (Fig. 4C, D). These findings indicate that HMGA1 promotes GBC progression by interacting with lncRNA TTN-AS1/miR-107 axis.

## Discussion

In recent years, many lncRNAs were identified to regulated GBC. For example, lncRNA-HGBC was upregulated in GBC and promoted GBC progression by regulating miR-502-3p/SET/AKT axis [27]. In the current study, lncRNA TTN-AS1 expression was also increased in GBC. Additionally, knockdown of lncRNA TTN-AS1 restrained cell viability and motility in GBC. Previous studies also suggested that lncRNA TTN-AS1 was upregulated in osteosarcoma and gastric cancer [28, 29]. Mechanistically, lncRNA TTN-AS1 was found to drive invasion and migration of lung adenocarcinoma cells [30]. LncRNA TTN-AS1 has been found to facilitate papillary thyroid cancer tumorigenesis by promoting cell proliferation [31]. These studies are consistent with our results, indicating that lncRNA TTN-AS1 serves as an oncogene in GBC progression.

Previous studies have shown that lncRNA TTN-AS1 is involved in the regulation of cancer by sponging miRNAs and mediating their functions. For instance, lncRNA TTN-AS1 sponged miR-376a-3p to promote colorectal cancer progression via upregulating KLF15 [32]. And Chen et al. reported that lncRNA TTN-AS1 promoted cell growth and metastasis in cervical cancer via miR-573/E2F3 [33]. Here, lncRNA TTN-AS1 was identified to promote cell viability and motility in GBC by sponging miR-107. It is worth noticing that miR-107 plays an important role in human cancers. For example, miR-107 was downregulated in esophageal squamous cell carcinoma and functioned as a tumor suppressor [34]. In our research, decreased miR-107 expression was also detected in GBC. And miR-107 overexpression suppressed cell viability and motility in GBC. It indicates that miR-107 acts a tumor inhibitor in GBC. Moreover, miR-107 has been reported to regulate tumorigenesis by competing with other lncRNAs, such as DLX6-AS1 and DLG1-AS1 [35, 36]. We found that lncRNA TTN-AS1 upregulation can abolish the inhibitory effect of miR-107 in this study, which is similar to previous studies. All these results imply that lncRNA TTN-AS1 promotes GBC development by acting as a ceRNA of miR-107.

Further, we found that HMGA1 was a direct target of miR-107. HMGA1 was upregulated in GBC and negatively correlated with miR-107 expression. Moreover, HMGA1 expression was positively regulated by lncRNA TTN-AS1 in GBC. Similar to our results, upregulation of HMGA1 has been identified in breast cancer and osteosarcoma [37, 38]. Functionally, HMGA1 was found to promote glioma cell growth in vivo and in vitro [39]. And knockdown of HMGA1 repressed cell proliferation and motility in bladder cancer [40]. Here, HMGA1 downregulation was also found to inhibit cell viability, migration, and invasion in GBC, suggesting that HMGA1 is a tumor promoter in GBC. Furthermore, TTN-AS1 upregulation or miR-107 downregulation can abolish the effect of HMGA1 in GBC. It shows that miR-107 can restrain GBC progression by downregulating HMGA1. Consistently, miR-142-3p has been demonstrated to function as a tumor suppressor in osteosarcoma by targeting HMGA1 [41]. Inversely, lncRNA TTN-AS1 was identified to exacerbate GBC malignancy by upregulating HMGA1, which has not been proposed in previous studies. Collectively, lncRNA TTN-AS1 acted as a tumor promoter in GBC by regulating miR-107/HMGA1 axis.

## Conclusion

Here, we demonstrated for the first time that lncRNA TTN-AS1 expression was increased in GBC. Furthermore, lncRNA TTN-AS1 acted as a tumor promoter in GBC by sponging miR-107 and upregulating HMGA1. In addition, miR-107 restrained GBC progression by downregulating HMGA1. However, there are still many shortcomings in this study. Therefore, further study still needs to be done in the future.

## Data Availability

The datasets used and/or analyzed during the current study are available from the corresponding author on reasonable request.
